# Laryngeal Cryptococcosis Associated With Inhaled Corticosteroid Use: Case Reports and Literature Review

**DOI:** 10.3389/fsurg.2017.00063

**Published:** 2017-11-13

**Authors:** Daniel Jun Yi Wong, Peter Stanley, Paul Paddle

**Affiliations:** ^1^Department of Otolaryngology, Head and Neck Surgery, Monash Health, Melbourne, VIC, Australia; ^2^Department of Infectious Diseases, St Vincent’s Hospital, Melbourne, VIC, Australia; ^3^Department of Surgery, Faculty Medicine, Nursing and Health Sciences, Monash University, Melbourne, VIC, Australia

**Keywords:** *Cryptococcus*, larynx, laryngitis, infection, fluconazole, antifungal

## Abstract

Laryngeal cryptococcosis is a rare clinical entity. There have been a limited number of case reports in the literature with no consensus regarding optimal management. This review contributes two additional case reports of immunocompetent patients with cryptococcal infection of the larynx in whom exposure to high doses of inhaled corticosteroids is proposed as a significant risk factor. Twenty cases were identified from review of the literature. All patients presented with hoarseness and a spectrum of microlaryngoscopic features, often mimicking laryngeal malignancy. The majority of cases were treated with systemic antifungal therapy, three cases had surgical excision alone, and another three had a combination of medical and surgical management. Risk factor modification, in the form of a reduction in inhaled corticosteroid was employed in the two new cases, and in some previously published cases. Risk factor modification, such as reduction of inhaled corticosteroid dose, in addition to oral antifungal agents can be effective in managing cryptococcal laryngitis.

## Introduction

Cryptococcal infections in general are rare. They are most commonly seen in the setting of immunosuppression with disseminated disease or occasionally as localized disease, namely meningitis, in the immunocompetent. Laryngeal cryptococcosis is a rare condition with only 20 previous cases reported. Here, we report two further cases of cryptococcal infection localized to the larynx. Both occurred in association with high-dose inhaled corticosteroid use. Laryngeal cryptococcosis, despite its rarity, is important as it may easily be confused with laryngeal malignancy, both macroscopically and histologically. These cases provide further evidence of inhaled corticosteroid local side effects and should strengthen the clinician’s resolve to use inhaled corticosteroids at their lowest effective dose.

## The Cases

### Case 1

A 66-year-old female presented with a 3-month history of progressive hoarseness. She did not have any other localizing or systemic symptoms. She had a history of asthma controlled on inhaled corticosteroids for 6 years. Her other history was of congenital pulmonary stenosis and lifelong allergic rhinosinusitis. She was a non-smoker and drank alcohol rarely. She had no known exposure to *Cryptococcus*.

Her inhaler medication, taken twice daily, was a combination powder product containing salmeterol (50 µg) and fluticasone (500 µg) per dose. In addition, she was taking an extra 500 µg twice daily of inhaled fluticasone, followed by inhaled nedocromil, 4 mg twice daily. She did not use a spacer or nebulizer but did rinse and gargle after each inhalation. She also took intra-nasal budesonide spray at a dose of 64 µg at night. She had never been on systemic steroid treatment.

**Figure 1:**
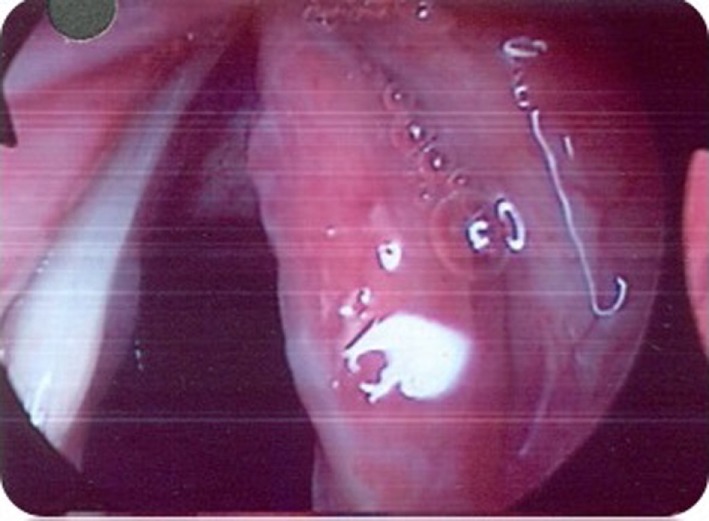
Case 1—Diffuse erythema and thickening of right true cord.

Stroboscopy revealed a markedly erythematous and thickened right true vocal fold with a marked reduction in the mucosal wave. The left true vocal fold was normal (see Figure 1).

**Figure 2:**
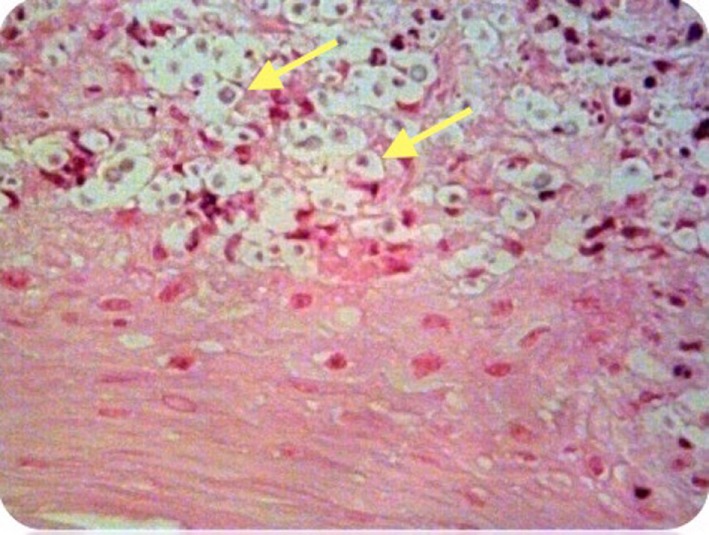
Case 1—Numerous encapsulated cryptococcal organisms (arrows) in vocal cord subepithelial stroma, H&E stain, 40×.

She underwent microlaryngoscopy and biopsy. Histological examination revealed collections of cryptococcal organisms lying within the superficial lamina propria, with an associated inflammatory infiltrate comprising abundant plasma cells, lymphocytes, and some histiocytes (see Figure 2). Her serum cryptococcal antigen was undetectable and her CXR unremarkable. She was HIV negative.

Treatment involved oral fluconazole, 200 mg orally, twice daily, and a reduction in her daily inhaled corticosteroid dose by half to 1,000 µg per day. Her budesonide spray was continued.

She was reviewed 7 weeks into treatment and had noted some improvement of her symptoms. At 6 months, her voice had returned to normal and repeat laryngoscopy was unremarkable. Her fluconazole was ceased after 6 months of treatment.

### Case 2

A 69-year-old female presented with a 1-month history of hoarseness and post-nasal drip. Her past history included a 10-year history of moderate persistent asthma which required a short course of oral prednisolone one to two times per year. In addition, she suffered from gastro-esophageal reflux disease and recurrent urinary tract infections.

Her asthma was treated with a combination preparation containing both salmeterol (25 µg) and fluticasone (500 µg) of which she was taking two puffs, twice per day. Thus a total dose of 2,000 µg per day of fluticasone was given for 3 months prior to her presentation. She always rinsed and gargled post inhaler, but did not use a spacer. It had been more than 7 months since her last course of oral prednisolone. She also used salbutamol nebules as required. Her only non-asthma medication was omeprazole at a dose of 20 mg twice daily.

She did not have any other systemic or localizing symptoms and had no other immunosuppressive medications or conditions. She was a non-smoker and had no known cryptococcal exposure.

**Figure 3:**
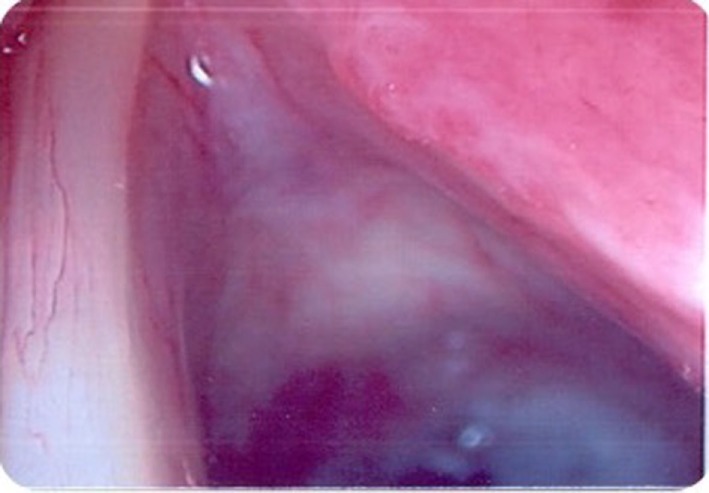
Case 2—Mucosal lesion right true vocal cord with focal erythroleukoplakia.

**Figure 4:**
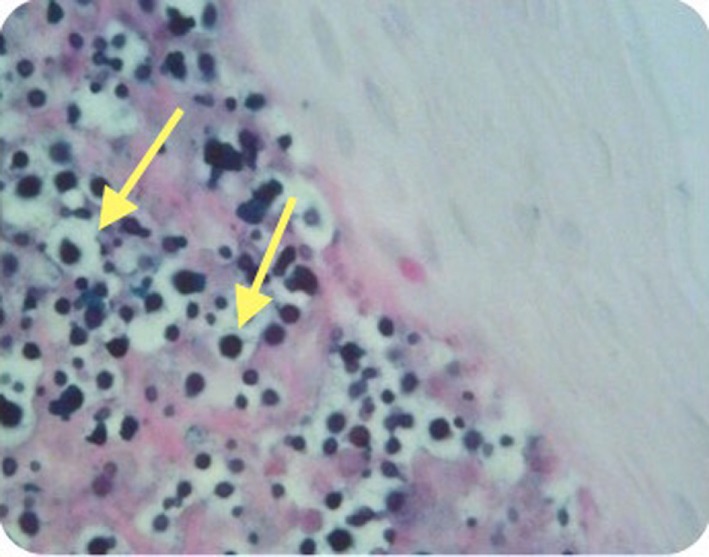
Case 2—Numerous encapsulated cryptococcal organisms in true cord subepithelial stroma (arrows), periodic acid-Schiff stain positive, 40×.

**Figure 5:**
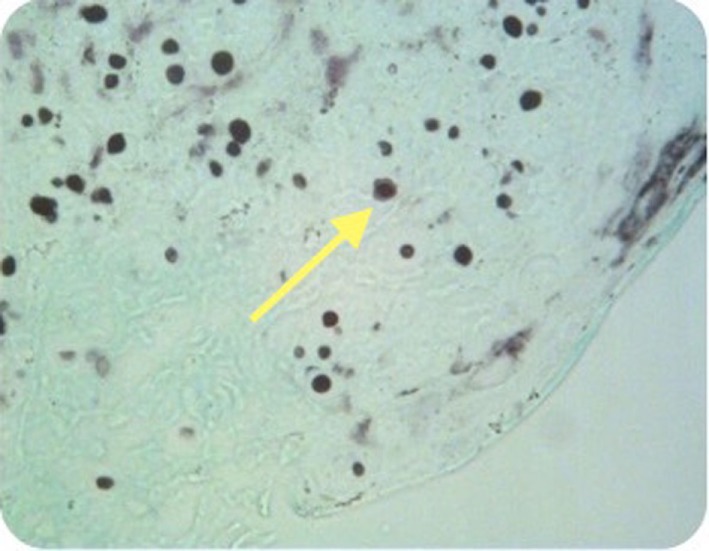
Case 2—Encapsulated cryptococcal organisms (arrow), methanamine silver stain positive, 40×.

At microlaryngoscopy her right true cord was found to be inflamed, with focal erythroleukoplakia (see Figure 3). Bronchoscopy and oesophagoscopy were normal. Histopathological examination of the biopsies of the involved fold revealed focal ulceration, underlying granulation tissue, and heavy lymphocyte and plasma cell infiltration. Aggregates of *Cryptococcus* organisms, as confirmed by periodic acid-Schiff and methanamine silver staining, lay in the surface inflammatory crust and within the subepithelial stroma (see Figures 4 and 5). There was no hyperplasia, cellular atypia, or signs of malignancy. Her cryptococcal serum antigen recorded a very low positive of 2 *via* the latex agglutination test. Her fasting glucose was unremarkable at 5 mmol/l. Her CXR was within normal limits, and her HIV test was negative.

Treatment involved oral antifungals and a reduction in her inhaled corticosteroid. She was instructed to reduce her inhaled fluticasone back to a daily maximum dose of 1,000 µg per day, and was placed on fluconazole at 200 mg twice a day.

She noticed improvement in her voice within weeks and after 5 months of fluconazole treatment her voice had returned to baseline. Stroboscopy 4 months into treatment revealed a near normal right true vocal fold with only slightly prominent vasculature, and near normal mucosal wave.

Her fluconazole was ceased after 8 months of treatment. Her voice remains normal off fluconazole, and at the lower dose of fluticasone.

## Discussion

*Cryptococcus neoformans* is an encapsulated yeast that lives as a saprobe in nature. While other types of *Cryptococcus* exist, it is only *C. neoformans* that is considered pathologically significant. It tends to be found in association with certain trees such as eucalypts and rotting wood. It is also consistently isolated from soil contaminated by guano from birds, especially pigeons, chickens, and turkeys. The route of infections remains somewhat unclear, but aerosolised particles that infect and disseminate after alveolar deposition in the lungs is thought to be the most likely portal of entry (1). At diagnosis, however, most cases do not have a defined epidemiological exposure or chest X-ray changes (1).

Given *Cryptococcus*’ relative ubiquity as an organism and rarity as a disease, it is thought that host factors, play an important role in the development of symptomatic disease. Between 15 and 30% of HIV/AIDS patients in sub-Saharan Africa will contract cryptococcal meningitis during the course of their illness (2). Other predisposing conditions for systemic disease include organ transplantation, lymphoproliferative disorders, hematological malignancy, systemic corticosteroid treatment, cirrhosis, and sarcoidosis (even without steroid treatment). An association with diabetes is not definitely known. A deficiency in cellular immunity seems to be the most important risk factor for cryptococcal disease (1). That said, one-third of patients lack any obvious immune deficit (1).

In immunosuppressed patients the pattern of disease tends to be disseminated with multiple sites being involved at the time of diagnosis, most commonly the lung and meninges, though the disease can involve practically any site. Similarly, mycotic infections of the larynx tend to arise, in immunocompromised hosts and also tend to occur as a result of dissemination of the organism, or at least contiguous spread, as is the case with laryngeal candidiasis, which usually coexists with oral candidiasis (3). That said, there are increasing reports of mycotic laryngitis with a normal immune status (*Candida*, aspergillosis, coccidiomycosis, histoplasmosis) (4).

The previously reported and current cases of laryngeal cryptococcosis are summarized in Table 1 with baseline characteristics listed in Table 2.

**Table 1:** Summary of cases of laryngeal cryptococcosis.

Reference	Age	Risk factors	*Cryptococcus* antigen	Gross lesion	Histopathology	Treatment and outcome
Reese and Coclasure (5)	47 M	Chicken manure	Positive	Marked laryngeal edema, glottis obstruction, multiple white raised exudative lesions	Pseudoepitheliomatous hyperplasia, granulomatous, budding yeast cells with large capsules, alcian blue+	Urgent tracheostomy, IV amphotericin for 1/12 (2 g)
						Pathological resolution 6/12 later

Smallman et al. (1989) (31)	31 F	Nil	Negative	Warty 0.5 lesion inferior to right true cord	Pseudoepitheliomatous hyperplasia, foreign body giant cells, mucicarmine and alcian blue+	Excisional biopsy. Patient refused further Rx
						Symptomatic resolution 1/12 post. Recurrence below right cord

Browning et al. (1992) (8)	46 M	HIV	Unknown	Right true cord erythema and edema from vocal process to anterior commissure	Granulomatous inflammation, methanamine silver and PAS+	Amphotericin B (120 mg for 4/7) then fluconazole
		Cryptococcal pneumonia				Symptomatic and pathological resolution

Frisch and Gnepp (1995) (12)	73 M	T2DM	Negative	Hyperemic fusiform mass on anterior 2/3 left false cord	Dense fibrous and granulation tissue, microcystic spaces with yeast structures, methanamine silver and mucicarmine+	Excisional biopsy
						Symptomatic resolution and no recurrence at 5/12

Kerschner et al. (1995) (14)	61 M	Oral prednisolone	Positive	Posterior commissure lesion, exophytic mass extending to arytenoids and false cords	Granulomatous, budding yeasts. mucicarmine+	Single dose 400 mg oral fluconazole, then 200 mg daily for 6/52Pathological and symptomatic resolution
		T2DM				
		Ex-smoker (35 pack-year history)				

Isaacson and Frable (1996) (18)	87 M	High-dose inhaled corticosteroid	Negative	Right anterior true vocal cord lesion—white and exudative	Pseudoepitheliomatous hyperplasia, marked submucosal inflammation. Methenamine silver and mucicarmine+	Fluconazole 400 mg/day for 2/12
		COPD, smoker				Cessation of inhaled steroids

Chongkolwatana et al. (1998) (10)	42 M	HIV+	Negative	Redness and irregularity of right anterior vocal cord and entire left vocal cord. Cords mobile, decreased amplitude and absence of mucosal wave on left cord	Pseudoepitheliomatous hyperplasia. Mucicarmine+	Fluconazole 400 mg/day for 8/52. Pathological resolution 9/12 from treatment. Symptomatic resolution
		Previous pulmonary TB				

McGregor et al. (13)	60 M	T2DM	Unknown	Right anterior true cord—verrucous lesion	Granulomatous inflammation, giant cell formation. Pseudoepitheliomatous hyperplasia, methanamine silver and mucicarmine+	Fluconazole 6/52
		Ex-smoker		Decreased right cord mobility		Symptomatic resolution. No evidence of pathological recurrence
		Tobacco chewer				

Nadrous et al. (2004) (16)	55 M	Inhaled corticosteroid	Negative	Right true cord—anterior leukoplakia and whole cord erythema	Squamous hyperplasia	Itraconazole 200 mg BD for 6/52, followed by Fluconazole 400 mg/daily for 10/52
		Intermittent systemic steroids for asthma			Acute and chronic histiocytic inflammation	Symptomatic and pathological resolution

Bamba et al. (2005) (21)	68 F	Smoking (50 pack-year history)	Unknown	Smooth sphenoid cystic mass in superomedial surface of right vocal cord. Normal vocal cord mobility bilaterally	2.5 subepithelial cystic specimen, containing small round monomorphic fungal bodies; alcian blue+, methenamine silver+	Surgical excision
						Symptomatic resolution post excision

Zeglaoui et al. (2005) (11)	65 F	HIV+	Positive	Infected budding lesion of laryngeal vestibule	Yeasts surrounded by capsules, consistent with *Cryptococcus neoformans*. Methenamine silver+	Amphotericin B (0.7 mg/kg/day) for 3/52 then Fluconazole 400 mg/day for 6 months
				Tumor-like with mobile cords		Pathological resolution. Death 11 months from diagnosis of AIDS from meningoencephalitis

Joo et al. (2009) (17)	82 F	Inhaled steroid	Positive	Edematous masses (right > left). Swelling and ulcerative-type masses on bilateral false vocal folds. Granulomas seen on posterior true vocal folds	Giemsa (GMS) and alcian blue stain+	Itraconazole for 6/52, Fluconazole for 10/52, further 2/12 of oral fluconazole. 585 nm pulsed dye laser
		Systemic corticosteroid for COPD				Incomplete pathological resolution at 4 months, 2× biopsy-confirmed residual laryngeal lesions. Near complete pathological resolution at 7 months with some symptomatic improvement

Gordon et al. (2010) (9)	64 M	Inhaled corticosteroid	Unknown	Patches of leukoplakia around the vocal cords with irregular subglottic mucosal margins	Pseudoepitheliomatous hyperplasia. Granulomatous inflammation. Methenamine silver and mucicarmine+	Fluconazole 400 mg/daily for 10/12
						Pathological resolution

Gordon et al. (2010) (9)	44 M	HIV+	Unknown	Bilateral thick, hyperemic vocal cords with subtle right cord irregularity. Thick anterior right vocal cord	Granulomatous inflammation, positive staining	Oral fluconazole for 3/12
		Hep C+				Symptomatic resolution
		Smoker				

Gordon et al. (2010) (9)	79 F	Inhaled corticosteroids	Negative	Bilateral vocal cord thickening	Crytpcoccus, mild inflammatory response, bilateral vocal cord thickening	Daily Fluconazole for 6/12
						Complete resolution

Chang et al. (6)	53 M	Exposure to pigeons	Negative	Mass on right posterior vocal cord	Squamous hyperplasia with acute and chronic inflammation, methenamine silver+, mucicarmine+	Fluconazole 400 mg daily for 6/52
						Symptomatic and pathological resolution

Mittal et al. (2013) (19)	58 M	Inhaled corticosteroid history of camping under eucalyptus trees one year prior	Negative	Congested red vocal cords. Irregular red lesion	Vocal fold squamous mucosa inflammation, thinned, and partially ulcerated. Alcian blue, methenamine silver+	Fluconazole 500 mg daily for 8/52

Bergeron et al. (2015) (20)	78 F	Inhaled corticosteroid	Negative	Bilateral whitish vocal cords	Hyperplasia, inflammation. Leukoerythroplakia of right vocal cord. alcian blue, mucicarmine+	Fluconazole 100 mg/daily for 4/52, fluconazole 100 mg/daily for 3/52, fluconazole 400 mg/daily for 15/52. Inhaled corticosteroids decreased. Symptomatic and pathological resolution

Jeng et al. (7)	71 F	Inhaled corticosteroids	Unknown	Exophytic lesions on posterior cord and right false vocal cord. White exophytic lesion on right vestibular fold, bilateral arytenoids and bilateral true vocal folds	Necrotic debris, inflamed squamous mucosa and fungal lesions on right medial arytenoid	Fluconazole 100 mg/daily for 2/52, fluconazole for 6/12. Surgical debulking
		Exposure to bird droppings				Symptomatic resolution at 4/12, pathological resolution at 11 months

Tamagawa et al. (2015) (15)	82 F	Systemic corticosteroid Salazosulfapyridine 500 mg daily	Positive	White exudative irregular lesion on right arytenoid	Pseudoepitheliomatous hyperplasia, severe submucosal inflammation. Methenamine silver+	Excision biopsy. Fluconazole 200 mg/daily for 182 days. Reduced dosage of corticosteroid over 4/12. Pathological resolution

Current case 1	66 F	High-dose inhaled corticosteroids	Negative	Erythematous right true cord	Cryptococcoma with inflammatory infiltrate	Reduction of inhaled fluticasone to 1,000 µg/day. Fluconazole 200 mg BD for 6/12
		Intra-nasal steroid				Symptomatic and pathological resolution

Current case 2	69 F	High-dose inhaled corticosteroids	Positive	Erythematous and thickened right true vocal cord	Cryptococcoma and infilammatory infiltrate, methenamine silver+	Reduction of inhaled fluticasone to 1,000 µg/daily
		Intermittent systemic steroid				Fluconazole 200 mg BD for 8/12
						Symptomatic and pathological resolution

**Table 2:** Baseline characteristics for 20 previously reported cases of laryngeal cryptococcosus.

Age range (years); mean	31–87; 59
Male: female ratio	12:8
**Symptoms^a^ (*N*, %)**	
Hoarseness	20 (100)
Cough	4 (20)
Dyspnea	1 (5)
**Predisposing factors^a^ (*N*, %)**	
HIV/AIDS	4 (20)
Diabetes mellitus	3 (15)
Exposure to birds	3 (15)
Smoking	5 (25)
Systemic corticosteroids	3 (15)
Inhaled corticosteroid	8 (40)

*^a^Patient may have more than one symptom or predisposing factor*.

Of the previously reported cases, only three had possible cryptococcal exposure. One patient had exposure to chicken manure (5), another had exposure to pigeons (6) and bird droppings prior to presentation (7). Several patients had a history of immunosuppresion: four with significant systemic immunosuppression in the form of HIV/AIDS (8–11); three patients with diabetes mellitus (12–14); and one patient was steroid-dependent and on immunosuppressant medication for rheumatoid arthritis (15). Importantly, 8 of the 20 cases (i.e., 40%), were using inhaled corticosteroids (7, 9, 16–20). Two of these patients were also on systemic steroids as a confounder for obstructive airways disease (16, 17). Our cases describe a further two immunocompetent patients with isolated laryngeal cryptococcosis who were exposed to inhaled corticosteroids. These cases further suggest that inhaled corticosteroids, particularly in high doses, are a potential risk factor for developing isolated laryngeal cryptococcosis in immunocompetent patients.

In immunocompetent patients with isolated laryngeal disease, the role of localized immunosuppression and disruption of the laryngeal mucosal barrier is important. Factors described as causing such a disruption include previous radiotherapy, gastro-esophageal reflux, traumatic intubation, smoking, and inhaled corticosteroids (3). Five of the reported patients had a history of smoking (9, 13, 14, 18, 21). Previous case reports have emphasized local immunosuppression from inhaled high-dose corticosteroids as a risk factor for laryngeal cryptococcal disease (9, 18–20). Our cases have similarities to those reported elsewhere in the literature with the exception of a stronger link to the use of high-dose inhaled corticosteroids.

Inhaled corticosteroid likely creates a localized immunosuppression within the larynx which along with its observed local irritation effect (22) causes an impairment of the epithelial barrier and allows the ubiquitous *Cryptococcus* to colonize and infect *via* direct inoculation. Studies suggest that up to 90% of inhaled drug is deposited in the upper airway of patients using inhalant devices, and it is this deposition that accounts for the local side effects of inhaled corticosteroids (23). Interestingly, descriptions of similar mycotic infections of the lungs, in immunocompetent patients are rare.

All of the reported patients using high-dose inhaled corticosteroids, including our two cases, presented with sub-acute onset dysphonia and a dry cough. Dysphonia is reported in up to 50% of patients using inhaled corticosteroids. The pathogenesis of steroid inhaler-related dysphonia is variable, but commonly caused by local irritation in the larynx from deposited inhalant. This irritation is seen across drug and propellant types (22), though dose and frequency seem to have an impact (22, 24). Laryngeal candidiasis can cause dysphonia or present as altered taste and is seen in around 5–10% of inhaled corticosteroid users (22, 25, 26). Adductor palsies of the vocal cords are another cause of dysphonia and thought to develop due to localized myopathy due to laryngeal corticosteroid deposition (22, 25, 26). Fluticasone is implicated as the most common cause of local steroid side effects such as dysphonia and oral candidiasis. Although this may merely reflect its widespread use (22, 24, 27–29), it is the most potent of the inhaled corticosteroids with the greatest receptor affinity and longest half-life (24, 27, 30). One study reported more than double the rate of oral candidiasis in those taking fluticasone compared with beclomethasone (29).

The differential diagnosis for the macroscopic appearance of the laryngeal cryptococcosis includes other fungal infections and malignancy. This is especially true in patients presenting with painless progressive dysphonia. This differential is echoed histologically where the typical pathological appearance of pseudoepitheliomatous hyperplasia of the squamous mucosa, and granulomatous inflammation in the submucosal region is seen in a number of conditions, including other mycotic infections (due to *Candida, Histoplasma, Blastomyces, Coccidioides*, or *Paracoccidiodes* species) (3), granular cell tumor, and early squamous cell carcinoma. This is further complicated by the fact that there is often a low yield of yeast cells in the biopsy, and these may be missed if not specifically looked for. This problem is compounded by the rarity of laryngeal cryptococcosis as practitioners may fail to consider the condition.

Treatment was varied among the published cases. Most cases in the literature were medically managed with oral antifungal agents; however, three cases had surgical excisions (12) and three had a combination of both (7, 15, 17). Fifteen cases received a minimum of 6–8 weeks of oral fluconazole treatment. One patient refused treatment following initial surgical excisional biopsy (31). One patient received a month of intravenous amphotericin (2 g total) (5). One HIV/AIDS patient received 4 days of intravenous amphotericin followed by maintenance fluconazole (8). Another with HIV had amphotericin for 3 weeks then oral fluconazole for another 6 months (11). Interestingly, two immunocompetent patients achieved cure with excisional biopsy alone (12, 21). Of the non-HIV patients who were treated, all remained disease free on prolonged follow-up. Most had taken weeks to months for their voices to normalize.

Importantly, we believe risk factor modification (by means of dose reduction in inhaled corticosteroid) is a useful adjunct to medical management, especially in patients taking high doses. On diagnosis, patients should be commenced on antifungal treatment without delay.

Review of the literature has shown that while 8 of the 20 published cases described inhaled corticosteroid as a potential risk factor, only two cases included some description of the dose (16, 18). Of these eight cases, only two described reduction or cessation of inhaled steroid as part of their management. Certainly, the remainder of the cases that did not describe any dose reduction still had symptomatic or pathological resolution after appropriate treatment. However, the lack of dose description makes it difficult to infer that the inhaled steroids were causing local laryngeal immunosuppression in all cases.

At present, there is uncertainty surrounding the evidence for dose-reduction alone in the setting of high-dose inhaled steroids in immunocompetent hosts with cryptococcal laryngitis. The decision for dose reduction should be made on a case-by-case basis, based on the severity of the patient’s pulmonary status. A change to ciclesonide, a relatively new inhaled corticosteroid could also be of potential benefit in treating laryngeal cryptococcosis. It is an inactive pro-drug, with a smaller particle size, which is converted to an active metabolite in the airways, with potentially fewer upper airway adverse effects. A recent systematic review comparing ciclesonide to other inhaled corticosteroids in the treatment of asthma in children, however, found no difference in adverse effects (25). Both our cases were inhaling doses exceeding the maximum recommended dose. Dose reduction in addition to oral fluconazole led to successful resolution.

## Conclusion

Laryngeal cryptococcosis, despite its rarity, is an important condition to diagnose, and as these two additional cases demonstrate, can occur in immunocompetent patients with possible local immunosuppression due to high dose inhaled corticosteroids. With correct diagnosis, risk factor modification (including inhaled steroid reduction), and oral antifungal treatment, it is a curable condition. It is especially important to be aware of the condition as a differential to early malignancy and other causes of inhaled steroid-related dysphonia.

## Author Contributions

All Authors were involved in the write-up of the manuscript, review, and editing. Dr Stanley contributed the clinical details of the two cases for discussion.

## Conflict of Interest Statement

The authors declare that they have no financial or other conflicts of interest in relation to this research and its publication.
